# Re-examining the importance of mask-wearing at mass gathering events

**DOI:** 10.1016/j.lanepe.2022.100423

**Published:** 2022-05-29

**Authors:** Michio Murakami

**Affiliations:** Center for Infectious Disease Education and Research (CiDER), Osaka University, 2-8 Yamadaoka, Suita, Osaka 565-0871, Japan

I read with great interest the study by Suñer et al. on the association between mass gathering events and risk of the coronavirus disease 2019 (COVID-19).^1^ This study assessed the infection risk associated with the events based on a sophisticated control-matched design and analyzed risk factors for infection through a questionnaire survey of event participants. The latter analysis showed that 74.7% of respondents wore masks at all or most of the time during the event, and that mask-wearing was a protective factor for infection (odds ratio = 0.64 [95% confidence interval: 0.54–0.77]). Masks are not only effective in reducing the risk of acquiring infections among exposed individuals, but also in preventing the spread of infection by viruses emitted from those already infected.^2^ The finding in this study related to the former effect; however, it is also important to evaluate the effectiveness of masks with respect to the latter. For example, what is the difference in the mask-wearing ratio among the uninfected and already infected participants at the time of the event? Also, is there any difference in the risk of post-event infection between participants who accompanied infectors who wore masks and those who accompanied infectors who did not wear masks? With the worldwide relaxation of mask-wearing in public, this study is of great value in that it prompts a re-examination of the significance of the effect of mask-wearing in public, especially at mass gathering events. I would be grateful for the authors’ opinions on this point.

## Declaration of interests

MM has attended the new coronavirus countermeasures liaison council jointly established by the Nippon Professional Baseball Organization and Japan Professional Football League as experts without any rewards.
